# Association between Frequency of Going Out and Psychological Condition among Community-Dwelling Older Adults after the COVID-19 Pandemic in Japan

**DOI:** 10.3390/healthcare10030439

**Published:** 2022-02-25

**Authors:** Suguru Shimokihara, Michio Maruta, Yasuaki Akasaki, Yuriko Ikeda, Gwanghee Han, Taishiro Kamasaki, Keiichiro Tokuda, Yuma Hidaka, Yoshihiko Akasaki, Takayuki Tabira

**Affiliations:** 1 Department of Rehabilitation, Medical Corporation, Nissyokai, Minamikagoshima Sakura Hospital, Kagoshima 890-0069, Japan; 2 Doctoral Program of Clinical Neuropsychiatry, Graduate School of Health Science, Kagoshima University, 8-35-1, Sakuragaoka, Kagoshima 890-8544, Japan; tai.pt.ft@gmail.com; 3 Department of Rehabilitation, Medical Corporation, Sanshukai, Okatsu Hospital, 3-95, Masagohonmachi, Kagoshima 890-0067, Japan; m.maru0111@gmail.com (M.M.); hidakayuma@icloud.com (Y.H.); 4 Visiting Researcher, Faculty of Medicine, Kagoshima University, Kagoshima 890-8544, Japan; hans11057@gmail.com; 5 Department of Occupational Therapy, School of Health Sciences, Faculty of Medicine, Kagoshima University, 8-35-1, Sakuragaoka, Kagoshima 890-8544, Japan; akaaki@m3.kufm.kagoshima-u.ac.jp (Y.A.); yuriko@health.nop.kagoshima-u.ac.jp (Y.I.); tabitaka@health.nop.kagoshima-u.ac.jp (T.T.); 6 Department of Neuropsychiatry, Kumamoto University Hospital, 1-1-1 Honjo Chuo-ku, Kumamoto 860-8556, Japan; 7 Department of Rehabilitation, Medical Corporation, Gyokushoukai, Kirameki Terrace Healthcare Hospital, Kagoshima 892-0824, Japan; gomyway.k.t@icloud.com; 8 Master’s Program of Health Sciences, Graduate School of Health Sciences, Kagoshima University, 8-35-1, Sakuragaoka, Kagoshima 890-8544, Japan; aka0805yfw1@yahoo.co.jp; 9 Department of Rehabilitation, Tarumizu Chuo Hospital, Kagoshima 891-2124, Japan

**Keywords:** community-dwelling older adults, COVID-19, epidemiology, frequency of going out, psychological condition

## Abstract

Background: The psychological condition and frequency of going out (FGO) of community-dwelling older adults after the spread of COVID-19 will provide insights for supporting the daily lives of community-dwelling older adults. Going out is defined as moving from one’s own home to a place or region beyond one’s own home and is considered to reflect the daily and social life of community-dwelling older adults. This study investigates the relationship between the FGO and current psychological condition after the second wave of COVID-19 in community-dwelling older adults in Japan. Methods: This study adopted a self-administered questionnaire by mail. A total of 493 members of CO-OP Kagoshima were included in the analysis and divided into two groups according to the change in FGO. Multiple logistic regression analysis was conducted after a bivariate analysis to investigate the relationship between the FGO and psychological condition. Results: Significant differences were noted between the groups with decreased FGO and those with increased/unchanged FGO in general and pandemic-related psychological condition. Multiple logistic regression analysis showed a significant relationship between FGO and psychological condition, such as mental fatigue, not smiling as much as before, and anxiousness to go outside. Conclusion: Community-dwelling older adults may have experienced a decrease in the frequency of going out and a detrimental effect on their psychological condition after the COVID-19 pandemic. This finding may inform strategies to identify priorities for psychological approaches altered by COVID-19 to prevent confinement and stress in older adults.

## 1. Introduction

The first COVID-19 outbreak was reported in December 2019 [1] and continues to have a severe impact causing high morbidity and mortality worldwide. The World Health Organization declared COVID-19 as a pandemic in March 2020 [2]. Consequently, the Japanese government declared a nationwide state of emergency from 16 April to 25 May 2020 [3]. The first and second waves of COVID-19 were defined from January to May 2020 and from June to August 2020, respectively [4]. The Japanese government closed public and recreational facilities and demanded that the citizens of Japan stay at home during this period. In particular, the risk of severe disease due to COVID-19 is thought to be high in the older population [5,6], and many older adults are vulnerable to adverse health effects. In addition, on 28 March 2020, the government decided on the Basic Policies for Novel COVID-19 Disease Control and called on the public to thoroughly implement social distance policies that limit the frequency and intensity of contact with people [7]. Specifically, people should keep a distance of at least two meters from each other and avoid crowded places and unnecessary social gatherings. This public health provision has been reported to affect the physical and mental functional aspects of older adults. The time of physical activity of older adults in Japan is reported to decrease by approximately 30% during the emergency declaration compared with that before the spread of COVID-19 [8]. In addition, community-dwelling older adults were reported to be aware of the decline in their physical and mental strengths due to voluntarily refraining from going out due to COVID-19 [9]. Mental health professionals have expressed serious concerns about the deterioration of mental health during and after a pandemic [10], especially among older adults [11]. Previous studies have linked social isolation to an increased risk of depression and anxiety disorders in older adults [12], and the psychological impact of the COVID-19 pandemic on older adults is certainly an area of research that is rapidly expanding. In particular, older adults perceive moderate stress due to COVID-19 outbreaks [13]; impact on psychiatric hospitalization of the older adults in the early stages of the epidemic [14]; and decline in quality of life, self-perceived health, and well-being before and after the lockdown [15], and insomnia was reported in 24.6% [16]. Hence, it is clear that mental health support for older adults is necessary for living with COVID-19, which is still expanding. Thus, reports examining the relationship between psychological condition due to COVID-19 and the frequency of going out (FGO) of older adults are lacking although the number of reports examining various physical and psychological effects of COVID-19 on older adults is rapidly increasing.

Here, going out is defined as the movement from one’s own home to a place or region beyond one’s own home as reported in a previous study [17]. The FGO in older adults reflects their social activity (e.g., their roles inside and outside the home, leisure activities, and interactions with others) [18]. Moreover, reports are examining the frequency of going out and psychological aspects (e.g., the COVID-19 pandemic reduced the frequency of going out and impaired the quality of life and mental health) [19,20,21]. However, there are few reports investigating the association between the FGO and psychological conditions during the COVID-19 pandemic in community-dwelling older adults. Investigating the FGO among community-dwelling older adults and identifying psychological conditions associated with a decrease in the FGO due to the spread of COVID-19 infection may help in understanding the current psychological conditions of older adults during the spread of COVID-19 infection. Therefore, this study aims to investigate the relationship between the FGO and psychological condition of the community-dwelling older adults after the second wave of the COVID-19 in Japan.

## 2. Materials and Methods

### 2.1. Study Design

This was a cross-sectional study using a self-administered questionnaire by mail dropped in a mailbox.

### 2.2. Participants

**Figure 1:**
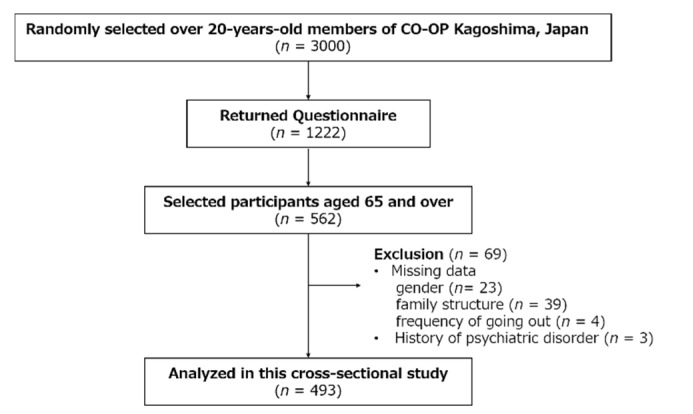
Flowchart of this study.

The participants of this study were recruited from the members of CO-OP Kagoshima, a consumer cooperative in Kagoshima Prefecture, Japan. The number of CO-OP members in Japan is >25 million, and CO-OP Kagoshima has approximately 310,000 members in 2018. A questionnaire titled “Questionnaire on changes in lifestyle due to the COVID-19” was sent to 3000 randomly selected CO-OP Kagoshima members ≥20 years old. The questionnaire included an enclosed reply envelope, and responses were collected via mail. The survey period was from September to October 2020. The sample size required to complete the survey was 220 people total (effect size = 0.3, α = 0.05, power = 0.95). These settings reference similar previous study designs [22,23,24,25]. Consequently, 1222 responses (recovery rate, 40.7%) were received. This study selected responses from those who were ≥65 years old from among the returned responses and excluded those with a history of psychiatric disorders (e.g., dementia and depression from the answers obtained) and incomplete answers. Finally, 493 participants (mean age 73.6, SD 7.0 years) were included in the analysis of this study. The flowchart of this study is shown in Figure 1.

### 2.3. Measurements

The self-administered questionnaire survey questions used in this cross-sectional study are discussed as follows. This questionnaire survey was conducted in Japanese. In addition, the questionnaire was validated by three skilled occupational therapists and one psychiatrist specializing in mental health.

#### 2.3.1. Social Demographics and Baseline Characteristics

This section asked the participants about their age, gender, family structure, and underlying disease. Age was self-reported, and the participants were asked to select an answer that applied for gender and family structure. For the question about underlying diseases, the participants were asked to select from the following items: hypertension, diabetes, hyperlipidemia, hyperuricemia, osteoporosis, cancer, cardiovascular disease, cerebrovascular disease, Parkinson’s disease, depression, dementia, collagen disease, spinal disease, thyroid disease, respiratory disease, osteoarthritis, ophthalmic disorders, hearing difficulty, fracture, and others. The number selected by the subject was used as the number of underlying diseases.

#### 2.3.2. Changes in Frequency of Going Out

This section first asked participants to compare the FGO before January 2019, the first wave of COVID-19 in Japan, with the FGO as of September–October 2020 (after the second wave of COVID-19 in Japan), the time of the survey. Participants were asked to select one answer based on their subjectivity (i.e., “Increased”, “Unchanged”, or “Decreased”).

#### 2.3.3. Psychological Condition Changes

Participants were asked in this section how their psychological condition changed before the COVID-19 spread (before January 2020) and after the second wave (after August 2020). The questionnaire items were developed by referring to the contents of the State–Trait Anxiety Inventory [26] and profile of mood states [27], which are assessment scales for anxiety and mood. We prepared questions that included characteristics of general and COVID-19 pandemic-related psychological conditions. In addition, to prevent differences in individuals’ specific prior experiences from influencing their judgments, care was taken to ensure that the psychological situations expressed in the items would be general psychological situations that would not remind most individuals of specific situations. In addition, items that can be judged to be compatible with Japanese social norms were given priority, while similar or special psychological situations were deleted. The total number of psychological situations (general and COVID-19 pandemic-related) was thus listed and used as survey items. Specifically, the following questions on psychological condition were included as items regarding general psychological condition: mental stress, mental fatigue, frustration, unmotivated, depression, bradyphrenia, sleeplessness, anorexia, emotional instability, anxiety, not smiling as much as before, increased amnesia, increased carelessness, increased restlessness, and increased nervousness. In addition, the following questions on psychological condition were included as items related to the COVID-19 pandemic: anxiousness to go outside, anxiousness to meet people, worry about not being able to see family, uneasiness on the information in the mass media, afraid of rumors, does not trust what others say, and being angry when someone is not wearing a mask. All questions were answered with yes or no, and medical terms that are not easily understood by the lay person were translated into words that can be understood and used. Factor Analysis by promax rotation with Kaiser normalization was conducted to check the construct validity of the survey items. The factors were classified into 8 categories in total (Table 1).

### 2.4. Statistical Analysis

Descriptive statistics were used to calculate the number of responses and percentages. Consequently, they were classified into two groups (those with decreased FGO and increased/unchanged FGO). To verify the reliability of this questionnaire, we calculated Cronbach’s α. A cross-tabulation table was then created from the answers obtained. Basic information and the percentage of responses to each question were then compared. For continuous variables, Student’s *t*-test or Mann–Whitney U-test was used based on the normality of the responses obtained. For the categorical variables, Pearson’s chi-square test was conducted. Effect size (ES) was estimated using Cohen’s d or Cramér’s V to assess the degree of difference. Multiple logistic regression analysis was conducted with decreased FGO as the dependent variable and the items that showed significant differences in the bivariate analysis as the independent variables. Potential covariates included age and gender. SPSS ver. 27.0 (IBM Corp., Armonk, NY, USA) was used for all analyses. The statistical significance level was set at *p* < 0.05. ES ≤ 0.2 was considered weak, 0.2 < ES ≤ 0.6 moderate, and ES > 0.6 strong.

### 2.5. Ethical Consideration

The study was conducted following the guidelines of the Declaration of Helsinki and approved by the ethical Committee in September 2020. Informed consent was obtained from all participants involved in the study.

### 2.6. Funding Sources

This research was funded by the CO-OP Insurance Support Project for Health Promotion.

## 3. Results

### 3.1. Characteristics of the Participants and Summary of Questionnaire

Table 2 shows the demographics of the participants. Among the 493 participants analyzed, 86% were women, and 69.8% had a decreased FGO. The decreased FGO group was significantly younger (t = –3.33, df = 491, *p* < 0.01, 95% confidence interval (CI), 0.13–0.52) and had a higher percentage of women (χ^2^ = 5.77, df = 1, *p* = 0.024) compared with the group with an increased/unchanged FGO. Cronbach’s α in this questionnaire was 0.66. Among the participants, 80.3% felt some changes in their psychological condition. In addition, 63.5% of the participants complained of some changes in the psychological condition in the general psychological condition questions, and a large percentage of them especially felt mental stress (28.6%), unmotivated (18.9%), and not smiling as much as before (18.3%). Furthermore, in terms of changes in psychological conditions related to the COVID-19 pandemic, 57.4% of the participants felt some psychological disorder. In particular, a large percentage of the participants answered anxiousness to go outside (28.4%), uneasiness about the information in the mass media (18.7%), and anxiousness to meet people (16.8%).

### 3.2. Bivariate Statistics for Psychological Condition Changed since before the COVID-19 Pandemic

Table 3 shows the change in psychological condition for each group. The group with decreased FGO compared with the group with increased/unchanged FGO showed the following changes in their general psychological condition: mental stress (χ^2^ = 14.61), mental fatigue (χ^2^ = 11.85), frustration (χ^2^ = 6.17), unmotivated (χ^2^ = 10.80), and not smiling as much as before (χ^2^ = 19.07). There was a significant difference in the following questionnaire items about COVID-19 pandemic-related psychological condition: anxiousness to go outside (χ^2^ = 23.55) and being angry when someone is not wearing a mask (χ^2^ = 7.35). Among the questionnaire items that were significantly different, a moderate effect size was observed for the item “anxiousness to go outside”.

### 3.3. Association between FGO and Mental Condition Changed since before the COVID-19 Pandemic

Table 4 shows the results of the multiple logistic regression analyses. The crude model showed that mental stress (odds ratio (OR): 1.69; 95% CI: 1.00–2.84, *p* = 0.047), mental fatigue (OR: 2.23; 95% CI: 1.10–4.53, *p* = 0.027), unmotivated (OR: 1.91, 95% CI: 1.02–3.57, *p* = 0.043), not smiling as much as before (OR: 2.98, 95% CI: 1.45–6.11, *p* = 0.003), and anxiousness to go outside (OR: 3.00, 95% CI: 1.74–5.15, *p* < 0.01) were significantly related to decreased FGO. Even after adjusting for potential covariates, mental fatigue (OR: 2.05, 95% CI: 1.00–4.20, *p* = 0.049), not smiling as much as before (OR: 3.43, 95% CI: 1.65–7.16, *p* < 0.01), and anxiousness to go outside (OR: 2.75, 95% CI: 1.59–4.74, *p* < 0.01) were significantly related to decreased FGO (adjusted model). The goodness of fit by the Hosmer–Lemeshow test were crude (χ^2^ = 7.82; *p* = 0.17) and adjusted (χ^2^ = 6.74; *p* = 0.57) models.

## 4. Discussion

A self-administered questionnaire survey of community-dwelling older adults (≥65 years old) was conducted to understand the relationship between psychological condition and FGO after the second wave of the COVID-19 in Japan. The results showed that the general and COVID-19-related psychological condition were significantly different among the groups classified by the change in FGO. Multiple logistic regression, after adjusting for potential covariates, showed a significant association between older adults who felt mental fatigue, did not smiling as much as before, and felt anxiousness to go outside and decreased FGO. These results suggest that it is important to understand that community-dwelling older adults with reduced FGO due to the COVID-19 pandemic may have unique psychological conditions and to develop preventive measures for mental health.

In the current study, 69.8% of the older adults reported a decrease in FGO. A similar consensus was reached in a previous study, which found that approximately two-thirds of community-dwelling older adults decreased their exercise behavior during declaring of emergencies [9], and 54.6% of those ≥85 years old reported going out less frequently [19]. The percentage of going out less frequently in the cohabitation group was similar to that in the previous studies [28]. The decrease in FGO among community-dwelling older adults may reflect a combination of factors, such as increased time at home [29], being a 65–75 years old who adheres to COVID-19 infection control measures [30], and decreased activity in daily life (e.g., shopping and eating out) due to social distancing policies [31]. However, the factors that the group with increased/unchanged FGO was significantly older, and the FGO has been low to begin with must be considered.

The participants experienced some symptoms suggesting that their psychological condition was affected by the COVID-19 social isolation restrictions and pandemic situation. Previous studies have shown that prolonged isolation and fear of the virus can lead to increased stress [32], and other trivial things that would normally not be of concern may become stressors during a pandemic. However, it should also be considered that the small effect sizes for the items that were found to be significantly different suggest other factors that affect the psychological condition and decrease in the FGO. For older adults, reduced FGO has been reported to be associated with risk of future disability [33,34], depressive symptoms, and cognitive complaints [35]. As with the association between the COVID-19 pandemic and psychological conditions, support may need to be considered for the secondary health effects of restricting older adults from going out.

Regarding the association between the FGO and psychological condition, the group with decreased FGO tended to feel more harm regarding items related to stress and items related to apathy. Furthermore, on items related to COVID-19, the lower FGO group revealed a significant association between feeling anxious about going out and decreased FGO. In addition, a significant association between obtaining information about COVID-19 from the media (e.g., television and the Internet) and high anxiety was found [36], suggesting that limiting media-viewing time and information sources may have a positive effect on mental health during the COVID-19 pandemic. In the results, the percentage of those who felt stressed and fatigued was significantly higher in the decreased FGO group, suggesting that some relationship may exist between vulnerability to stress and lower FGO in older adults. Thus, conducting long-term follow-up observations is necessary to examine the effects of psychological condition on health-related outcomes.

The Japanese government declared a nationwide state of emergency from 16 April to 26 May 2020. Specifically, public facilities and entertainment venues were closed, and people were asked to avoid crowded places and unnecessary social gatherings. This may have restricted the places where older adults could engage in social and recreational activities (e.g., community centers and karaoke boxes). In a previous Japanese national survey on social activities among older adults, it was reported that 14.7% of the older adults (≥60 years old) participated in social activities at least once a month [37]. Furthermore, the laughter generated during interpersonal interaction was assumed to also decrease because it has been reported that interpersonal interaction and social activities decreased after the COVID-19 epidemic [31]. Maintaining social activities while implementing basic infection control measures is necessary to support the participation of community-dwelling older adults in social activities after the spread of COVID-19. Specifically, supporting the social activities of older adults while taking basic infection-control measures (e.g., using remote intervention [38], wearing a mask [39], and keeping a 2-m distance [40]) is desirable.

This study has several limitations. First, mentioning the causal relationship between changes in psychological condition and changes in FGO is not possible because the research design is cross-sectional. Therefore, a longitudinal study needs to be conducted to confirm the causal relationship. Second, this study applied a simpler, non-standardized questionnaire to assess one’s FGO and psychological condition in emergency conditions related to COVID-19. This questionnaire was created with a psychiatrist specialized in mental health due to the particular conditions of the post-pandemic period. Cronbach’s α, while not high, is reasonable considering that it measures a more general and broader psychological condition. Further validation is needed to assure this approach. Moreover, the change in psychological condition and FGO was self-reported and retrospective. The current study has only investigated the participants’ subjective FGO and psychological conditions during the measurement period has changed compared to the pre-COVID-19 pandemic period. Therefore, this study cannot fully rule out recall bias although dementia and psychiatric disorders were excluded. Future work will need to focus on the baseline FGO and what factors would determine staying active during the COVID-19 pandemic. Third, the participants were members of CO-OP Kagoshima who were ≥65 years old and were not randomly selected. In addition, data on COVID-19 emergencies may vary depending on the region and the prevalence of infectious diseases; this study was conducted in Kagoshima, a prefecture located in the southern part of Japan, where the COVID-19 infection status is different compared to the metropolitan area. In particular, since the FGO in the winter season is expected to decrease in heavy snowfall areas, it is necessary to examine the seasonal effect in future studies. Fourth, the response rate was not high and may have been limited to participants with cognitive and physical functions who were able to answer the questionnaire survey completely and post it back to the mailbox. In order to eliminate regional bias in this study, the subjects were selected evenly from municipalities in Kagoshima Prefecture, and it was not possible to identify all individual respondents. Therefore, it was not able to calculate the response rate limited to the participants over 65 years old. Consequently, an unintended selection bias cannot be ruled out. The recruitment target should be further expanded to generalize the results of this study.

In conclusion, this study confirmed a significant association between psychological conditions and a decrease in FGO after the second wave of COVID-19 pandemic among community-dwelling older adults in Japan. This suggests that the community-dwelling older adults during the COVID-19 pandemic may have experienced a decrease in the FGO and a detrimental effect on their psychological condition, such as mental fatigue, less smiling, and anxiety about going outside. Thus, taking preventive measures against COVID-19 infection while considering the secondary health hazards and taking approaches and interventions to improve the psychological condition are expected.

## Figures and Tables

**Table 1:** Factor Analysis by promax rotation with Kaiser normalization.

	Factor ^a^							
	1	2	3	4	5	6	7	8
uneasiness about the information in the mass media	**0.66**	0.09	0.02	−0.14	−0.38	−0.12	0.02	0.19
increased nervousness	**0.66**	0.03	−0.06	0.22	−0.05	0.08	−0.32	−0.05
increased restlessness	**0.64**	−0.01	−0.09	−0.09	0.15	0.15	−0.11	0.01
anxiety	**0.51**	−0.10	0	0.09	0.01	−0.07	0.42	−0.13
mental fatigue	0.04	**0.80**	0.11	−0.19	−0.15	0.13	−0.11	0.04
sleeplessness	−0.05	**0.74**	−0.04	0.37	0.01	−0.29	−0.03	−0.01
depressed	0.27	**0.33**	−0.04	0.18	0.29	−0.12	0.15	0.02
emotional instability	0.05	**0.31**	0.04	−0.27	0.27	0.22	0.12	−0.26
anxiousness to meet people	−0.10	0.09	**0.83**	0.16	−0.1	0	0	0.02
anxiousness to go outside	−0.04	−0.09	**0.76**	0.04	0.06	−0.13	0.25	0.10
being angry when someone is not wearing a mask	0.05	0.25	**0.45**	−0.03	0.11	0.20	−0.21	−0.06
does not trust what others say	0.10	−0.10	0.10	**0.80**	0.05	0.11	−0.08	−0.07
increased carelessness	−0.10	0.15	0.08	**0.60**	−0.16	0.29	0.09	−0.03
frustration	−0.14	−0.12	−0.01	−0.01	**0.87**	−0.01	−0.09	−0.01
mental stress	0.19	0.09	0.02	−0.15	**0.41**	−0.08	0.23	0.13
bradyphrenia	−0.01	0	−0.04	0.17	0.07	**0.74**	−0.12	0.07
afraid of rumors	0.32	−0.18	0.08	0.13	−0.2	**0.5**	0.15	−0.11
increased amnesia	−0.17	0.23	−0.26	0.08	−0.18	**0.35**	0.34	0.09
worry about not being able to see family	−0.15	−0.09	0.07	−0.03	−0.04	−0.06	**0.82**	−0.15
not smiling as much as before	−0.04	0.03	0.05	−0.04	0.02	0.28	**0.41**	0.19
anorexia	0.01	0.08	0.06	−0.17	−0.11	−0.05	−0.13	**0.81**
unmotivated	0.04	−0.13	0	0.17	0.32	0.24	−0.02	**0.6**

Note: Kaiser–Meyer–Olkin value = 0.69; Bartlett’s test of sphericity; F = 231, *p* < 0.001. ^a^—Factor loadings indicate the strength of association between each variable and each factor, with a factor loading of ≥0.3 indicated in bold.

**Table 2:** Characteristics of the study participants.

Characteristic	Overall, *n* = 493	Increased/Unchanged FGO, *n* = 149	Decreased FGO, *n* = 344	ES	*p*-Value
Age ^†^	73.6 (7.0)	75.17 (7.25)	72.92 (6.72)	0.326	0.001 ^a^
Gender ^‡^				0.108	0.024 ^b^
male	68 (14%)	29 (19%)	39 (11%)		
female	425 (86%)	120 (81%)	305 (89%)		
Family structure ^‡^				0.019	0.666 ^b^
living alone	126 (26%)	40 (27%)	86 (25%)		
living with family	367 (74%)	109 (73%)	258 (75%)		
Number of underlying diseases ^§^	1 (0–7)	1 (0–7)	1 (0–6)		0.771 ^c^

Statistics presented: ^†^ mean (SD), ^‡^ n/N (%), ^§^ median (minimum–maximum). Statistical tests performed: ^a^ *t*-test; ^b^ chi-square test of independence, ^c^ Mann–Whitney U test. Abbreviation: FGO, frequency of going out; ES, effect size.

**Table 3:** Bivariate statistics for mental condition changed since before the COVID-19 pandemic.

Question Items	Overall, *n* = 493 ^†^	Increased/Unchanged FGO, *n* = 149 ^†^	Decreased FGO, *n* = 344 ^†^	ES	*p*-Value
General psychological condition					
Mental stress				0.172	<0.001
yes	141 (29%)	25 (17%)	116 (34%)		
Mental fatigue				0.155	<0.001
yes	79 (16%)	11 (7.4%)	68 (20%)		
Frustration				0.112	0.024
yes	25 (5.1%)	2 (1.3%)	23 (6.7%)		
Unmotivated				0.148	0.002
yes	93 (19%)	15 (10%)	78 (23%)		
Depressed				0.058	0.253
yes	57 (12%)	13 (8.7%)	44 (13%)		
Bradyphrenia				0.074	0.141
yes	42 (8.5%)	8 (5.4%)	34 (9.9%)		
Sleeplessness				0.079	0.111
yes	55 (11%)	11 (7.4%)	44 (13%)		
Anorexia				0.068	0.206
yes	20 (4.1%)	3 (2.0%)	17 (4.9%)		
Emotional instability				0.001	0.988
yes	10 (2.0%)	3 (2.0%)	7 (2.0%)		
Anxiety				0.079	0.111
yes	55 (11%)	11 (7.4%)	44 (13%)		
Not smiling as much as before				0.197	<0.001
yes	90 (18%)	10 (6.7%)	80 (23%)		
Increased amnesia				0.043	0.426
yes	56 (11%)	20 (13%)	36 (10%)		
Increased carelessness				0.017	0.853
yes	33 (6.7%)	9 (6.0%)	24 (7.0%)		
Increased restlessness				0.002	0.965
yes	13 (2.6%)	4 (2.7%)	9 (2.6%)		
Increased nervousness				0.022	0.799
yes	23 (4.7%)	8 (5.4%)	15 (4.4%)		
COVID-19 pandemic-related psychological condition					
Anxiousness to go outside				0.219	<0.001
yes	140 (28%)	20 (13%)	120 (35%)		
Anxiousness to meet people				0.084	0.084
yes	83 (17%)	18 (12%)	65 (19%)		
Worry about not being able to see family				0.062	0.218
yes	69 (14%)	16 (11%)	53 (15%)		
Uneasiness about the information in the mass media				0.014	0.562
yes	92 (19%)	25 (17%)	67 (19%)		
Afraid of rumors				0.032	0.725
yes	35 (7.1%)	12 (8.1%)	23 (6.7%)		
Does not trust what others say				0.024	0.180
yes	14 (2.8%)	7 (4.7%)	7 (2.0%)		
Being angry when someone is not wearing a mask				0.074	0.010
yes	72 (15%)	12 (8.1%)	60 (17%)		

Statistics presented: ^†^; n (%). Statistical tests performed: chi-square test of independence. Abbreviation: FGO, frequency of going out; ES, effect size.

**Table 4:** Association between frequency of going out and psychological condition changed since before the COVID-19 pandemic.

	Crude Model			Adjusted Model		
	Wald χ^2^	OR (95% CI)	*p*-Value	Wald χ^2^	OR (95% CI)	*p*-Value
Mental stress						
no ^a^	-	-	-	-	-	-
yes	3.94	1.69 (1.01–2.84)	0.047	3.07	1.60 (0.95–2.70)	0.080
Mental fatigue						
no ^a^	-	-	-	-	-	-
yes	4.90	2.23 (1.10–4.53)	0.027	3.89	2.05 (1.00–4.20)	0.049
Frustration						
no ^a^	-	-	-	-	-	-
yes	3.18	3.96 (0.87–18.00)	0.074	2.99	3.80 (0.84–17.28)	0.084
Unmotivated						
no ^a^	-	-	-	-	-	-
yes	4.10	1.91 (1.02–3.57)	0.043	3.59	1.84 (0.98–3.47)	0.058
Not smiling as much as before						
no ^a^	-	-	-	-	-	-
yes	8.85	2.98 (1.45–6.11)	0.003	10.80	3.43 (1.65–7.16)	0.001
Anxiousness to go outside						
no ^a^	-	-	-	-	-	-
yes	15.78	3.00 (1.74–5.15)	<0.001	13.17	2.75 (1.59–4.74)	<0.001
Being angry when someone is not wearing a mask						
no ^a^	-	-	-	-	-	-
yes	1.37	1.52 (0.75–3.08)	0.242	1.93	1.66 (0.81–3.40)	0.165
Gender						
Female ^a^	-	-	-	-	-	-
Male	-	-	-	2.18	0.65 (0.36–1.15)	0.140
Age	-	-	-	7.78	0.96 (0.93–0.99)	0.005

In each model, frequency of socialization set as a dependent variable; adjusted model: adjusted for age, gender. ^a^ denotes reference groups. Abbreviation: OR, odds ratio; CI, confidence interval.

## Data Availability

The data that support the findings of this study are available on request from the corresponding author, S.S. The data are not publicly available due to restrictions that their containing information that could compromise the privacy of research participants.
